# Treatment of hypertrophic lichen planus using deucravacitinib

**DOI:** 10.1016/j.jdcr.2024.06.013

**Published:** 2024-07-05

**Authors:** Siddhartha Sood, Geeta Yadav

**Affiliations:** a Temerty Faculty of Medicine, University of Toronto, Toronto, Ontario, Canada; b Division of Dermatology, Temerty Faculty of Medicine, University of Toronto, Toronto, Ontario, Canada; c Division of Dermatology, Women's College Hospital, Toronto, Ontario, Canada; d Probity Medical Research, Waterloo, Ontario, Canada; e FACET Dermatology, Toronto, Ontario, Canada

**Keywords:** case report, deucravacitinib, hypertrophic, JAK inhibitor, lichen planus, TYK2 inhibitor

## Introduction

Lichen planus (LP) is an immune-mediated lymphocytic skin condition with heterogeneous presentations including cutaneous, mucosal, follicular, and nail involvement.^1^ Hypertrophic LP represents a subtype of cutaneous disease with prominent pruritic, scaly, and hyperkeratotic papules.^1^ Despite often reflecting a recalcitrant and chronic disease course, there currently remains no US Food and Drug Administration-approved therapy for LP.^2^ Therefore, off-label therapy is often necessitated for refractory disease. Deucravacitinib, a selective tyrosine kinase 2 (TYK2) inhibitor, has received approval in several countries for the treatment of moderate-to-severe plaque psoriasis.^3^ TYK2 receptors are implicated in several cytokine pathways via the Janus kinase (JAK)-signal transducer and activator of transcription (STAT) pathway, including T-helper 1-specific mediators such as interleukin (IL)-12, which may be implicated in LP pathogenesis.^4^^,^^5^ Furthermore, evidence has suggested TYK2 inhibitors may have a more tolerable safety profile than other JAK inhibitors due to the increased selectivity of JAK-STAT blockade.^3^^,^^4^ Although there are emerging reports on the use of related JAK inhibitors for several subtypes of LP, no studies have evaluated the use of TYK2 inhibition for cutaneous LP.^6^ Herein, we report a case involving the novel use of deucravacitinib leading to successful resolution in a patient with hypertrophic LP.

## Case report

**Fig 1:**
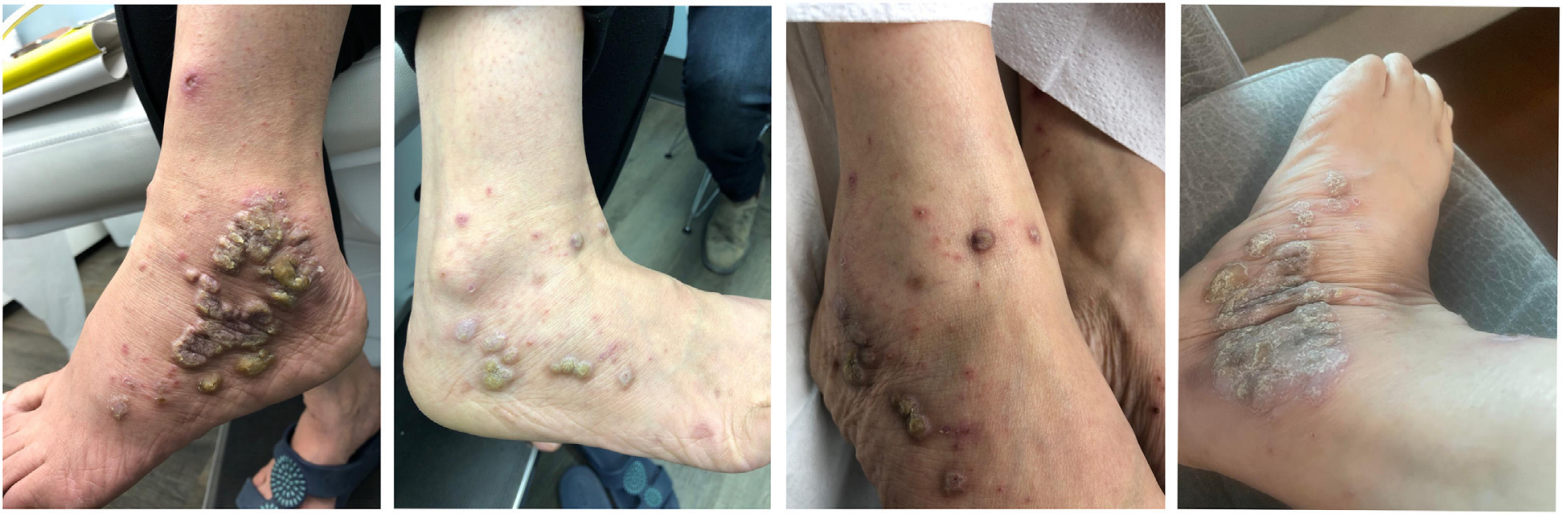
Baseline involvement of hypertrophic cutaneous lichen planus on the bilateral ankles and feet.

A 45-year-old female was referred to our dermatology practice with a prior 15-year history of plaque psoriasis, reported to be biopsy-proven and diagnosed in India. She presented with a pruritic, hyperkeratotic, and violaceous eruption on her bilateral ankles, wrists, back, and hips (Fig 1). She complained of itch and pain in her vulva for the last 1 year. She trialed topical calcipotriol/betamethasone dipropionate on these lesions for several weeks without significant improvement. A biopsy of the right ankle was performed with prominent histopathological findings including hyperkeratosis, irregular acanthosis, and hypergranulosis of the basal epidermis with dense lymphocytic infiltrates of the epidermal-dermal junction. This was in keeping with a diagnosis of cutaneous LP with hypertrophic morphological features.

**Fig 2:**
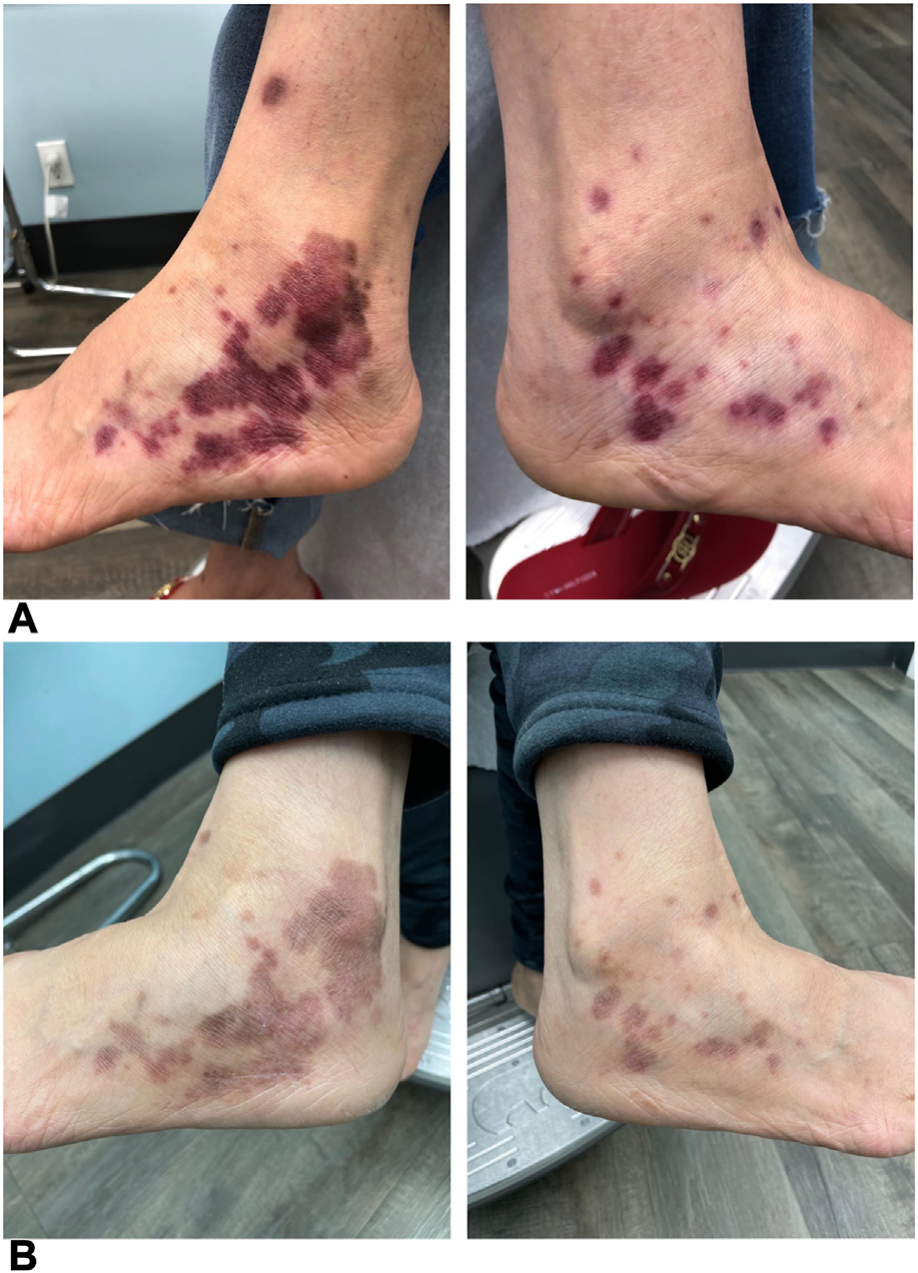
Successful resolution of hypertrophic lichen planus with deucravacitinib treatment. **A,** Significant improvement of the bilateral ankles with follow-up at 1.5 months. **B,** Complete clearance at 4.5 months.

On initial assessment, the patient was provided with 0.05% clobetasol propionate cream along with 5% salicylic acid for use; however, this did not improve the lesions. The patient declined intralesional triamcinolone acetonide due to concerns regarding pain and practicality of the multiple injections that would be required. Although conventional systemic therapy for LP was discussed, the patient was hesitant of the safety profile. Ultimately, the decision was made to initiate oral deucravacitinib off-label. Following baseline laboratory and bloodwork monitoring, she was started on 6 mg once daily dosing of deucravacitinib with permitted concomitant use of 5% salicylic acid and clobetasol propionate to affected areas under occlusion twice daily. On the first follow-up, 1.5 months later, significant improvement was seen with regression of hyperkeratotic plaques in affected areas (Fig 2, *A*). At this point, daily use of topicals was discontinued and the patient was advised to use clobetasol propionate and roflumilast for the feet and vulva, respectively, as needed for any flares. Although the patient briefly trialed discontinuation of deucravacitinib for 2 weeks, this led to a vulvar flare. While roflumilast was unable to clear the lesions, resolution of clinical symptoms was achieved at 4.5 months following deucravacitinib reinitiation and brief use of clobetasol propionate on the vulva (Fig 2, *B*). She has maintained this response when seen at the last follow-up, approximately 9 months from baseline. The patient was maintained on the initial dosing regimen during the entire treatment period with no reported or observed adverse events to date.

## Discussion

Although the pathogenesis of cutaneous hypertrophic LP remains unclear, recent studies have probed into the role of JAK/STAT signaling.^7^ A recent systematic review identified 56 LP patients receiving JAK inhibitor therapy, demonstrating complete resolution with upadacitinib (100%), baricitinib (25%), ruxolitinib (16.7%), and tofacitinib (10%).^5^ In contrast to these agents, deucravacitinib is selective for TYK2 receptors which are ubiquitously involved in the regulation of several cytokine pathways.^4^ In-vitro studies have demonstrated upregulated type I and type II interferons (IFNs) expression in both cutaneous and mucosal biopsies of LP.^8^, ^9^, ^10^ Furthermore, higher levels of dermal IL-12 expression and serum IL-23 have been implicated in patients with cutaneous LP.^6^^,^^7^^,^^10^ Therefore, TYK2 inhibition may provide a dual mechanistic effect to modulate LP inflammation by directly inhibiting type I IFNs and indirectly downregulating mediators of type II IFNs such as IL-12/IL-23. These findings may provide a rationale for the favorable response we observed with deucravacitinib use in the patient described and may indicate a particular role for TYK2 in the regulation of LP inflammation.

This study is limited by a lack of standardized disease severity measures for LP and limited generalizability. Nonetheless, to our knowledge, this is the first case in the literature regarding the use of deucravacitinib for hypertrophic cutaneous LP. This report represents the novel utility of TYK2 inhibition for this condition, demonstrating a sustained response and favorable long-term tolerability, supporting the indication for large-scale prospective and/or retrospective studies to further inform clinical application.

## Conflicts of interest

Dr Yadav reports personal fees from Johnson & Johnson Innovative Medicine, AbbVie Inc., Amgen, Aralez, Cipher, Arcutis, Bausch Health, Bristol Myers Squibb, Galderma, Incyte, Leo, Novartis, Paladin, Pfizer, Sanofi-Regeneron, Sun Pharma, and UCB outside the submitted work. Mr Sood has no conflicts of interest to declare.
